# Acute Finnish sauna heat exposure induces stronger immune cell than cytokine responses

**DOI:** 10.1080/23328940.2026.2645467

**Published:** 2026-03-31

**Authors:** Ilkka H.A. Heinonen, Tiia Koivula, Maija Hollmén, Jaakko Immonen, Setor K. Kunutsor, Sirpa Jalkanen, Jari A. Laukkanen

**Affiliations:** a Turku PET Centre, University of Turku and Turku University Hospital, Turku, Finland; b The UKK Institute for Health Promotion Research, Tampere, Finland; c MediCity Research Laboratory, University of Turku, Turku, Finland; d Department of Medicine, Wellbeing Services County of Central Finland, Jyväskylä, Finland; e Section of Cardiology, Department of Internal Medicine, Max Rady College of Medicine, Rady Faculty of Health Sciences, University of Manitoba, Saint Boniface Hospital, Winnipeg, Manitoba, Canada; f Department of Physiology and Pathophysiology, Rady Faculty of Health Sciences, University of Manitoba, Winnipeg, Manitoba, Canada; g Institute of Clinical Medicine, Department of Medicine, University of Eastern Finland, Kuopio, Finland

**Keywords:** Heat, stress, sauna bathing, immune cells, white blood cells, cytokines, health

## Abstract

Regular exposure to Finnish sauna bathing (FSB) has been associated with reduced morbidity and mortality. This study aimed to examine the acute effects of FSB on immune cell mobilization, circulating cytokines, and their associations with changes in body temperature. A total of 51 adults − 27 women (mean age 50 ± 9 years, body mass index (BMI) 27 ± 5 kg/m^2^) and 24 men (mean age 50 ± 10 years, BMI 27 ± 3 kg/m^2^) – were exposed to a 30-minute session of acute FSB at a temperature of + 73°C. Venous blood samples were collected at baseline, immediately after and 30 minutes after the FSB and analyzed for immune cells and 37 cytokines. Subjects were allowed to drink water throughout. FSB increased body temperature from 36.4 ± 0.5°C to 38.4 ± 0.7°C, without altering plasma volume. Total white blood cell (WBC) count rose significantly and remained slightly elevated 30 minutes post-sauna in women. Neutrophil and lymphocyte counts increased immediately after the FSB but returned to baseline after 30 minutes, whereas MXD cells (monocytes, eosinophils, basophils) remained elevated. The levels of only two cytokines changed significantly. Although only a few correlations were observed between changes in immune cells and cytokines, 18 significant associations were identified between changes in body temperature and circulating cytokines – particularly immediately post-sauna – but not with WBC changes. Thus, a 30-minute session of acute FSB induces immune cell mobilization. The observed associations between changes in body temperature and circulating cytokines suggest that sauna-induced heat stress, along with immune activation, may partly mediate the health benefits of FSB.

## Introduction

Increasing temperatures and especially extreme heat waves [1] due to climate change threatens the nature and well-being of humans and other animals. Although adaptations also occur [2], this is because heat has profound influence on physiology, including humans [3–7]. While chronic heat stress is generally harmful to health, short-term exposure to acute heat – even at high intensities – can produce beneficial effects [8]. Finnish sauna bathing represents one such acute heat stress exposure, where individuals are subjected to relatively dry air at high temperatures (typically 70–100 °C) for 10–30 minutes per session. This traditional practice has been associated with a range of benefits, including reductions in the risk of hypertension [9], sudden cardiac death [10,11], venous thromboembolism [12], stroke [13], dementia and Alzheimer’s disease [14] and psychosis [15,16]. Additionally, sauna use has been linked to lower risks of respiratory conditions such as pneumonia, chronic obstructive pulmonary and other respiratory diseases [17–22]. It may also mitigate the adverse effects of low socioeconomic status [23], improve physical fitness [24], and ultimately contribute to decreased overall morbidity and mortality [25]. Although the physiological mechanisms mediating these health effects have been partially explored – particularly those related to cardiovascular function [26,27] – the exact pathways remain incompletely understood [26].

The protective effects of heat therapies may, in part, be mediated through immunological responses. In our previous population-based studies, regular Finnish sauna bathing was associated with lower levels of systemic inflammation as indicated by reduced C-reactive protein (CRP) levels [28,29]. Additionally, acute sauna exposure has been linked to increased white blood cell (WBC) counts [30]. However, the specific effects of sauna-induced heat stress on leukocyte subpopulations and circulating cytokines – which play key roles in mediating immune responses – have not been fully characterized. Therefore, the aim of the present study was to investigate the acute effects of Finnish sauna bathing on immune cell mobilization and a comprehensive panel of circulating cytokines in middle-aged men and women, including their interrelationships and associations with changes in body temperature.

## Methods

### Participants

Participants of the study were recruited from the city of Jyväskylä, Central Finland region, through the local out-of-hospital community health care center. Study participants were asymptomatic with at least one cardiovascular risk factor, such as a history of smoking, dyslipidemia, hypertension, obesity, diabetes, or family history of coronary heart disease. Participants with any form of acute or preexisting cardiovascular disease were excluded from the study. A total of 51 participants were finally included in the present study. Given the challenge in recruiting naive or non-sauna users to sauna studies in Finland because the majority of the population use sauna regularly at home, our study sample was based on sauna users. Prior to study entry, participants were provided with information about the research purposes and measurement procedures and were screened by a cardiac specialist. The study design and protocol were approved by the institutional review board of the Central Finland Hospital District ethical committee, Jyväskylä, Finland (Dnro 5 U/2016). All study participants provided written informed consent.

### Baseline assessments and clinical examination

Baseline data collection as well as clinical evaluation were conducted on separate days prior to the experiment. Assessment of demographics, lifestyle factors (e.g. smoking, physical activity, sauna bathing habits), prevalent diseases, and regular use of medication were based on a detailed self-reported questionnaire which was checked by a cardiologist during the screening. Medical history, physical examination, and resting electrocardiogram were assessed during the screening visit. Body mass index (BMI) was calculated by dividing weight in kilograms by the square of height in meters.

### Finnish sauna heat stress exposure

The traditional Finnish sauna with dry air and relatively high temperature was used as our exposure. It consisted of a typical Finnish sauna bathing session which lasted for 30 minutes. However, there was a short, two-minute shower after the first 15 minutes. Sauna bathing sessions took place between 9.00–11.00 hrs on the specified study days. The sauna rooms were sex specific, and all participants wore their own swim suits during the sauna sessions. Only one participant was allowed in the sauna bath at a time. Sauna temperature was set at 80° Celsius and this was controlled and monitored by internal temperature sensors designed by Harvia Oy, Finland. Temperature assessment was continuous with the use of a 2-channel thermometer in the sauna room and the data was collected during experiment. The temperature sensor also monitored the humidity of the sauna room. Based on the overall data collected, the mean ± standard deviation (SD) temperature was 73 ± 2°C with a relative humidity of 10–20%. Study participants were monitored and supervised by a physician and were allowed to leave the sauna at any time they felt any discomfort. All participants underwent the recommended sauna protocol without any problems. Participants were given 500 ml of still water at room temperature to drink during the entire sauna session and the 30-minute recovery period after sauna. The recovery period was for 30 min which involved resting in a designated relaxing waiting lounge (mean temperature 21 °C). Body temperature was measured from the ear for each participant using tympanic thermometry.

### Blood samples and measurements of white blood cells and cytokines

Non-fasting blood samples were taken about 2 hrs prior to sauna sessions. Participants were instructed to abstain from strenuous physical activity 24 h before the blood samples were taken. Venous blood samples were collected by a qualified laboratory technician from the antecubital vein in the sitting position, using sterile needles and were collected into serum and plasma tubes (BD Vacutainer, Plymouth. UK). Whole blood samples were stored for 10 min before being centrifuged at 3500 rpm (Megafuge, Heraeus, Germany) and serum samples were stored at −80°C until analysis. Blood samples for basic hematological parameters and WBC counts were analyzed on site by Sysmex KX 21 (Sysmex Co., Kobe, Japan) analyzer. Comprehensive 37-kit multiplex cytokine assay was performed by multiplex cytokine profiling. Cytokines from stored serum were measured using a Bio-Plex Pro Human Inflammation Panel 1 (Bio-Rad, cat. #171AL001M) and a Bio-Plex 200 System (Bio-Rad) [31] according to the manufacturer’s instructions.

The change in plasma volume (PV) during and after sauna bout was taken into consideration and calculated with the following formula: ΔPV = (Hb_pre_*(1-Hct_post_))/(Hb_post_*(1-Hct_pre_))-1 [32]. WBC counts and cytokines were adjusted to reflect the exercise-induced shifts in the plasma volume with the following formula: WBC_corrected_ = WBC_uncorrected_*(1+ΔPV), and respectively for cytokines [32].

## Statistical analysis

Data were presented as means ± (SDs). Absolute values (means) of laboratory variables before and after sauna were analyzed for analysis of variance (ANOVA) test (sex and time and their interaction as main factors) and correlations with Pearson correlation. In an additional analysis subjects were divided into three different study group based on their weekly sauna use and responses (changes) in studied variables were analyzed by one-way ANOVA. The level for significance was set at *p* < 0.05. All statistical analyses were carried out with SAS 9.4 version statistical analyses program.

## Results

**Figure 1.**
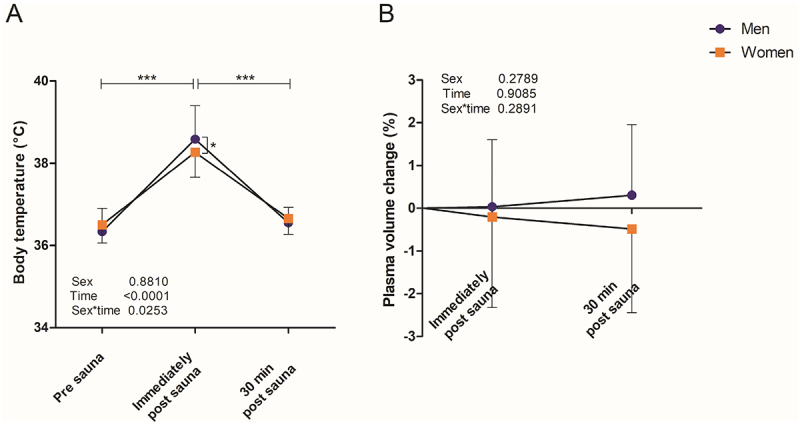
Body temperature (A) and plasma volume change (B) from baseline to immediately post-sauna and 30-minute post-sauna exposure.

A total of 51 apparently healthy adults − 27 women (mean age 50 ± 9 years, BMI 27 ± 5 kg/m^2^) and 24 men (mean age 50 ± 10 years, BMI 27 ± 3 kg/m^2^) participated and were exposed to 30 minutes of acute Finnish sauna bathing at + 73°C temperature. Characteristics of the subjects are presented in detail in Table 1. Temperature in the ear was increased significantly from the beginning to the end of the sauna session, but plasma volume did not change on average as a response to sauna-induced heat stress (Figure 1). Blood-based markers were still corrected by individual plasma volume change to account for individual responses and slight changes not causing any hemoconcentration-induced effects of the results.

*p < 0.05 between men and women, ***p < 0.001 as compared to pre and 30-minute post sauna exposure.

**Table 1.** Characteristics of the subjects.

Variable		N
Age, years	50.1 (9.5)	51
Weight, kg	79.0 [68.1, 93.3]	48
Body mass index, kg/m^2^	26.5 [23.8, 29.9]	48
Sex, female	27 (52.9)	51
Hypertension, yes	5 (10.0)	50
Type 2 diabetes, yes	1 (2.0)	50
Sauna bathing frequency (at home)		50
Not at all	16 (32.0)	
Less than once a week	16 (32.0)	
2–3 times a week	16 (32.0)	
4–7 times a week	2 (4.0)	
Sauna bathing duration, min	30 [20,40]	49
Sauna temperature, C°	70 [65, 80]	49

Data are mean (sd), median [Q1, Q3] or n (%).

**Figure 2.**
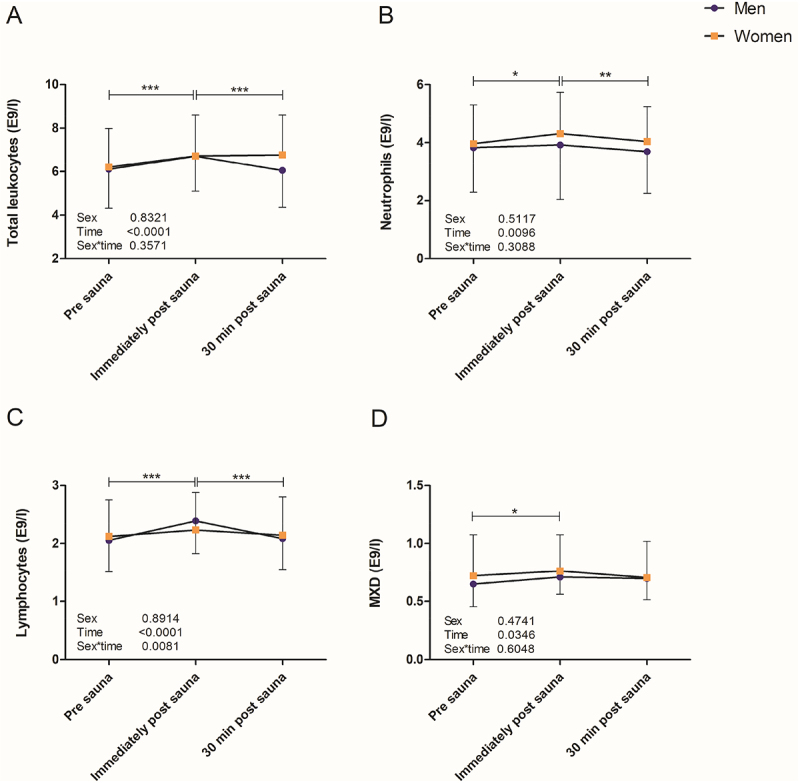
Total leukocyte (A), neutrophil (B), lymphocyte (C) and MXD cells (D) from baseline to immediately post-sauna and 30-minute post-sauna exposure.

Hemoglobin and hematocrit were significantly lower and thrombocytes tended to be higher in women and all these variables increased in response to sauna bathing similarly in both sexes (Table 2). Total WBC count increased significantly and remained slightly elevated 30-minute post sauna in women (Figure 2(A), Table 2). Neutrophils, lymphocytes and MXD cells (monocytes, eosinophils and basophils) were increased significantly immediately post-sauna, but reduced to baseline levels 30-minute post-sauna, while MXD cells (monocytes, eosinophils and basophils) remained elevated also 30-min after the sauna exposure (Figure 2(B–D), Table 2). While there were these changes in the total blood cell counts, proportions of the WBCs did not change (Table 2).

*<0.05, **<0,01,***p < 0.001 as compared to pre and 30-minute post sauna exposure.

**Table 2.** Blood biomarker profile before and after sauna bathing.

	Pre	Post	30 min Post	p-values				
				sex time sex*time				
Men	149 (8)	154 (9)***	150 (8)†††					
Hemoglobin, g/l				**<0.0001 < 0.0001 < 0.0001**				
Women	133 (9)**^^^**	133 (8)**^^^**	132 (9)**^^^**					
Men	45 (2)	46 (3)***	45 (3)†††					
Hematokrit, %				**<0.0001 < 0.0001 < 0.0001**				
Women	41 (3)**^^^**	41 (3)**^^^**	40 (3)*,**^^^**					
Men	232 (45)	249 (49)*	235 (47)†††					
Trombocytes, E9/l				0.0841 **<0.0001 0.0482**				
Women	264 (50)	272 (56)***	250 (48)†††					
Men	6.1 (1.8)	6.7 (1.6)***	6.2 (1.7)†††					
Total leukocytes, E9/l				0.8321 **<0.0001** 0.3571				
Women	6.2 (1.8)	6.7 (1.9)***	6.5 (1.8)*,†					
Men	3.8 (1.5)	3.9 (1.9)	3.7 (1.5)					
Neutrophils, E9/l				0.5117 **0.0096** 0.3088				
Women	4.0 (1.3)	4.3 (1.4)**	4.0 (1.2)†					
Men	2.1 (0.5)	2.4 (0.6)***	2.1 (0.5)†††					
Lymphocytes, E9/l				0.8914 **<0.0001 0.0082**				
Women	2.1 (0.6)	2.2 (0.6)*	2.2 (0.7)					
Men	0.65 (0.2)	0.72 (0.1)*	0.71 (0.2)					
MXD, E9/l				0.4741 **0.0346** 0.6048				
Women	0.72 (0.4)	0.75 (0.3)	0.78 (0.3)					
Men	57 (8)	57 (8)	57 (7)					
Neutrophils, %				0.7011 0.3270 0.3972				
Women	57 (5)	55 (13)	56 (5)					
Men	32 (7)	33 (8)	32 (7)					
Lymphocytes, %				0.8174 0.6994 0.6482				
Women	32 (5)	32 (6)	33 (5)					
Men	8.5 (4.4)	7.1 (4.7)	7.6 (4.7)					
MXD, %				0.0563 0.5057 0.1189				
Women	4.9 (3.5)	5.4 (3.9)	6.4 (3.6)					

Data presented as mean (SD). MXD=monocytes-basophils-eosinphils mixed.

*p<0.05, **p<0.01, ***p<0,001 change from baseline; †p<0.05, †††p<0.001 change from post; ^^p<0,01, ^^^p<0,001 between sexes at time point.

Concentration of two cytokines (sCD30/TNFRSF8 and Pentraxin-3) were significantly reduced and one cytokine (MMP-2) tended to be increased (*p* = 0.052) by the sauna session (Table 3). Many associations (*n* = 18) were evident between the change in body temperature and circulating cytokines (Table 4), particularly immediately after the sauna exposure, but not with the changes in WBCs (Table 4). Cytokines whose change correlated positively with the change in ear temperature were IFN-a2, IFN-b, IFN-y IL-2, IL-8, IL-11, IL-12 (p40), IL-19, IL-20, IL-22, IL-28A/IFN-lambda2, IL-29/IFN-lambda1, IL-35, MMP-1, TSLP, TWEAK/TNFSF12 and cytokines whose change correlated negatively with the change in the ear temperature were Chitinase 3-like1 and gp130/sIL-6Rb (Table 4).

**Table 3.** Blood cytokine levels (units in all variables pg/ml) before and after sauna bathing.

	Pre	Post	30 min Post	p-values				
				sex time sex*time				
Men	99,748 (27747)	103,073 (28430)	109,165 (22901)					
APRIL/TNFSF13				0.1759 0.3465 0.3948				
Women	91,853 (37901)	92,872 (37053)	92,295 (38081)					
Men	8340 (2489)	8617 (2263)	8749 (2121)					
BAFF/TNFSF13B				0.9078 0.5474 0.2956				
Women	8450 (3199)	8675 (3250)	8266 (3245)					
Men	382 (277)	351 (198)	401 (273)**††**					
sCD30/TNFRSF8				0.0712 **0.0454** 0.2395				
Women	291 (120)	273 (112)	280 (118)					
Men	82,883 (32389)	79,079 (29637)	86,333 (28559)					
sCD163				0.5039 0.4682 0.5107				
Women	78,198 (38022)	76,215 (35252)	76,274 (35919)					
Men	7206 (4231)	7044 (3331)	7862 (3478)					
Chitinase 3-like1				0.1486 0.2399 0.2872				
Women	5852 (3781)	5910 (3721)	5918 (3902)					
Men	32,878 (12405)	30,470 (9743)	36,370 (7947)					
gp130/sIL-6Rb				0.1654 0.1524 0.3082				
Women	29,742 (13723)	28,524 (14428)	29,311 (13094)					
Men	61 (22)	62 (24)	56 (23)					
IFN-a2				**0.0264** 0.2017 0.8276				
Women	49 (17)**^**	49 (18)**^**	46 (15)					
Men	31 (15)	32 (20)	33 (16)					
IFN-b				**0.0231** 0.9883 0.7172				
Women	25 (17)	24 (15)	22 (10)**^**					
Men	38 (9)	38 (11)	37 (10)					
IFN-y				**0.0064** 0.5354 0.9926				
Women	32 (9)**^**	32 (11)**^**	30 (9)**^**					
Men	6.6 (2.9)	6.4 (4.3)	6.6 (4.6)					
IL-2				**0.0397** 0.9218 0.9316				
Women	4.9 (3.1)	4.7 (2.9)	4.5 (3.7)					
Men	6079 (2284)	5743 (1905)	6458 (1879)					
sIL-6Ra				0.6936 0.3150 0.4377				
Women	6082 (2668)	5716 (2576)	5791 (2614)					
Men	25 (15)	24 (15)	22 (12)					
IL-8				0.1148 0.1874 0.8929				
Women	20 (10)	18 (12)	17 (12)					
Men	13 (5)	13 (4)	13 (4)					
IL-10				0.3351 0.6539 0.8387				
Women	12 (5)	11 (6)	13 (5)					
Men	3.2 (1.8)	3.5 (2.8)	2.8 (2.2)					
IL-11				0.1216 0.4747 0.6564				
Women	2.6 (1.7)	2.4 (1.7)	2.2 (1.8)					
Men	84 (36)	90 (40)	76 (32)					
IL-12 (p40)				0.2195 0.0970 0.2930				
Women	80 (35)	71 (37)	68 (31)					
Men	3.1 (5.6)	3.0 (4.9)	2.9 (5.9)					
IL-12 (p70)				0.4168 0.2998 0.9087				
Women	2.3 (2.8)	2.0 (2.8)	1.9 (3.0)					
Men	21 (7)	22 (9)	21 (7)					
IL-19				0.1368 0.3792 0.9883				
Women	19 (6)	20 (6)	18 (6)					
Men	163 (38)	160 (41)	159 (38)					
IL-20				**0.0144** 0.3864 0.8095				
Women	143 (36)	138 (42)**^**	131 (34)**^**					
Men	111 (412)	112 (426)	134 (523)					
IL-22				0.9655 0.2272 0.1541				
Women	29 (21)	29 (23)	28 (24)					
Men	66 (18)	62 (20)	69 (18)					
IL-26				0.6521 0.9065 0.0510				
Women	63 (31)	65 (31)	60 (28)					
Men	25 (22)	29 (21)	37 (36)					
IL-27				0.5810 0.3544 0.7051				
Women	23 (16)	31 (17)	28 (14)					
Men	90 (73)	86 (51)	92 (95)					
IL-28A/IFN-lambda2				0.0570 0.7673 0.6670				
Women	63 (16)	62 (19)	61 (19)**^**					
Men	150 (49)	154 (58)	144 (56)					
IL-29/IFN-lambda1				0.0952 0.3272 0.9552				
Women	128 (46)	136 (59)	122 (51)					
Men	205 (85)	213 (97)	184 (92)					
IL-35				0.0602 0.3484 0.4706				
Women	166 (73)	162 (78)	161 (50)					
Men	2417 (659)	2344 (792)	2219 (823)					
MMP-1				**0.0044** 0.2891 0.9721				
Women	1955 (630)**^**	1847 (694)**^**	1777 (692)**^**					
Men	10,552 (3941)	10,680 (3482)	11,687 (4384)					
MMP-2				0.3594 0.0520 0.3798				
Women	11,632 (5428)	12,471 (5483)	12,422 (6075)					
Men	10,370 (4479)	10,700 (4725)	10,912 (4261)					
MMP-3				**0.0023** 0.9723 0.0737				
Women	7635 (3168)**^**	7208 (3110)**^^**	7092 (3042)**^^^**					
Men	3173 (1142)	3260 (1092)	3638 (1174)					
Osteocalcin				0.4497 0.0558 0.0741				
Women	3756 (2026)	3672 (2288)	3740 (2324)					
Men	61,016 (18774)	57,989 (15686)	65,524 (15087)					
Osteopontin				0.9541 0.2262 0.2644				
Women	63,260 (29245)	60,998 (31394)	61,397 (30929)					
Men	247 (112)	228 (90)	236 (80)					
Pentraxin-3				0.2848 **0.0492** 0.5765				
Women	300 (179)	258 (145)*	266 (152)					
Men	1364 (413)	1380 (365)	1494 (256)					
sTNF-R1				0.0653 0.6804 0.1609				
Women	1218 (571)	1187 (561)	1158 (528)					
Men	3127 (1196)	2968 (999)	3351 (1044)					
sTNF-R2				0.1220 0.4970 0.1184				
Women	2762 (1405)	2664 (1367)	2543 (1238)					
Men	163 (37)	173 (51)	164 (41)					
TSLP				**<0.0001** 0.4262 0.1200				
Women	125 (30)**^^^**	116 (37)**^^^**	115 (34)**^^^**					
Men	306 (70)	311 (80)	313 (78)					
TWEAK/TNFSF12				0.8572 0.3803 0.0843				
Women	323 (82)	295 (103)	300 (98)					

Data presented as mean (SD).

*p<0.05, **p<0.01 change from baseline; †p<0.05, ††p<0.01 change from post.

^p<0.05, ^^p<0,01, ^^^p<0,001 between sexes.

**Table 4.** Correlations between change in body temperature and white blood cell counts and cytokine levels.

	Post-Pre			30 min Post-Pre				
	no adjustement	adjusted with sex	adjusted with BMI	no adjustement		adjusted with sex	adjusted with BMI	
Total leukocytes	−0.08	−0.11	0.01	−0.01		0.18	0.17	
Neutrophils	0.07	0.11	0.07	0.15		0.29	0.28	
Lymphocytes	0.07	−0.15	0.10	−0.08		0.15	0.15	
MXD	0.09	−0.07	−0.13	0.03		−0.14	−0.16	
APRIL/TNFSF13	−0.06	−0.07	−0.06	−0.18		−0.22	−0.18	
BAFF/TNFSF13B	0.06	0.05	0.04	−0.05		−0.08	−0.04	
sCD30/TNFRSF8	−0.05	−0.02	−0.07	−0.12		−0.15	−0.12	
sCD163	−0.19	−0.19	−0.23	−0.24		−0.26	−0.24	
Chitinase 3-like1	−0.29*	−0.29*	−0.32*	−0.16		−0.19	−0.16	
gp130/sIL-6Rb	−0.05	−0.04	−0.08	−0.29		−0.33*	−0.30	
IFN-a2	0.41**	0.42**	0.44**	0.02		−0.00	0.01	
IFN-b	0.30*	0.30	0.33*	−0.39*		−0.39*	−0.39*	
IFN-y	0.35*	0.36*	0.35*	−0.17		−0.17	−0.18	
IL-2	0.29	0.31*	0.28	−0.22		−0.24	−0.22	
sIL-6Ra	−0.14	−0.15	−0.16	−0.07		−0.10	−0.06	
IL-8	0.33*	0.33*	0.35*	0.03		0.03	0.03	
IL-10	0.29	0.26	0.28	−0.11		−0.13	−0.11	
IL-11	0.38*	0.36*	0.42**	−0.21		−0.20	−0.20	
IL-12 (p40)	0.44**	0.41*	0.45**	−0.05		−0.06	−0.06	
IL-12 (p70)	0.28	0.27	0.28	−0.00		−0.04	−0.02	
IL-19	0.49***	0.51***	0.50***	0.04		0.04	0.04	
IL-20	0.29*	0.29*	0.29	0.01		−0.00	0.01	
IL-22	0.28*	0.26	0.28	−0.38**		−0.41**	−0.40**	
IL-26	−0.16	−0.11	−0.18	0.13		0.11	0.15	
IL-27	0.28	0.29	0.30	0.26		0.25	0.26	
IL-28A/IFN-lambda2	0.47***	0.52***	0.47**	−0.17		−0.17	−0.17	
IL-29/IFN-lambda1	0.27	0.29*	0.28	−0.07		−0.07	−0.07	
IL-35	0.43**	0.43**	0.49***	−0.31		−0.31	−0.31	
MMP-1	0.41**	0.41**	0.42**	−0.08		−0.08	−0.08	
MMP-2	−0.07	−0.02	−0.09	−0.05		−0.06	−0.05	
MMP-3	−0.05	−0.14	−0.04	−0.03		−0.06	−0.02	
Osteocalcin	0.04	0.00	0.02	0.01		−0.03	0.01	
Osteopontin	−0.23	−0.23	−0.26	−0.03		−0.05	−0.02	
Pentraxin-3	0.04	0.00	0.01	−0.07		−0.10	−0.07	
sTNF-R1	−0.07	−0.10	−0.10	−0.20		−0.25	−0.20	
sTNF-R2	−0.16	−0.16	−0.19	−0.19		−0.23	−0.19	
TSLP	0.41**	0.34*	0.43**	−0.20		−0.23	−0.20	
TWEAK/TNFSF12	0.29*	0.21	0.30*	−0.27		−0.32	−0.27	

Significant *p*-values;*<0.05, **<0.01, ***<0.001.

Very few correlations were detected between changes in immune cells and cytokines (Tables 5 and 6). Further, when subjects were divided into three different groups based on their weekly sauna use habits (Table 1, group 1 not at all, group 2 less than once per week, and group 3, 2–3 times per week or more), there were no differences in responses in any of the studied variables (plasma volume or temperature change, or changes in total white blood cells or its sub-populations, or cytokines, data not shown).

**Table 5.** Correlations between change in white blood cell counts and cytokine levels (post-pre).

	WBC	Neutrophils	Lymphocytes	MXD
APRIL/TNFSF13	−0.16	0.00	−0.03	0.16
BAFF/TNFSF13B	−0.18	0.06	−0.03	0.13
sCD30/TNFRSF8	−0.20	0.22	−0.15	0.17
sCD163	−0.15	−0.01	−0.03	0.23
Chitinase 3-like1	−0.02	0.03	0.08	0.30
gp130/sIL-6Rb	−0.02	0.03	−0.02	0.24
IFN-a2	−0.02	0.20	0.17	−0.22
IFN-b	0.00	0.23	0.05	−0.10
IFN-y	−0.20	0.31	−0.11	−0.02
IL-2	−0.24	0.23	−0.26	0.05
sIL-6Ra	−0.08	0.07	−0.04	0.13
IL-8	−0.09	0.26	−0.05	−0.01
IL-10	−0.03	0.02	0.12	−0.33*
IL-11	0.05	0.16	0.08	−0.17
IL-12 (p40)	−0.03	0.22	0.11	−0.07
IL-12 (p70)	0.17	0.20	0.30	−0.20
IL-19	0.03	0.42**	0.12	−0.00
IL-20	−0.08	0.20	0.11	−0.21
IL-22	−0.32*	0.10	−0.33*	0.32*
IL-26	−0.22	−0.26	−0.06	−0.06
IL-27	0.14	0.31	0.05	−0.39
IL-28A/IFN-lambda2	−0.10	0.57***	−0.12	0.08
IL-29/IFN-lambda1	0.06	0.46**	0.06	0.05
IL-35	0.22	0.34*	0.22	−0.02
MMP-1	−0.19	0.21	−0.14	−0.10
MMP-2	−0.18	−0.04	−0.02	0.15
MMP-3	−0.09	0.05	0.11	−0.00
Osteocalcin	−0.07	0.18	0.09	0.05
Osteopontin	−0.04	−0.05	0.01	0.10
Pentraxin-3	−0.12	−0.03	−0.01	0.14
sTNF-R1	−0.12	0.19	−0.07	0.24
sTNF-R2	−0.12	0.25	−0.11	0.27
TSLP	0.02	0.31*	0.17	0.01
TWEAK/TNFSF12	−0.06	0.10	0.10	0.00

Significant *p*-values; *<0.05, **<0.01, ***<0.001.

**Table 6.** Correlations between change in white blood cell counts and cytokine levels (30 min post-pre).

	WBC	Neutrophils	Lymphocytes	MXD
APRIL/TNFSF13	−0.02	0.09	−0.00	−0.27
BAFF/TNFSF13B	0.14	0.24	0.06	−0.09
sCD30/TNFRSF8	0.11	0.20	0.10	−0.11
sCD163	0.11	0.20	0.08	−0.19
Chitinase 3-like1	0.10	0.14	0.09	−0.07
gp130/sIL-6Rb	0.12	0.11	0.21	−0.07
IFN-a2	−0.22	−0.09	−0.25	−0.31
IFN-b	−0.08	0.05	−0.06	−0.24
IFN-y	0.01	0.17	−0.18	−0.25
IL-2	0.24	0.34	0.06	−0.24
sIL-6Ra	0.07	0.14	0.07	−0.18
IL-8	−0.14	0.07	−0.16	−0.31
IL-10	0.06	0.15	0.01	−0.15
IL-11	−0.18	−0.15	0.07	−0.19
IL-12 (p40)	−0.23	−0.05	−0.20	−0.35*
IL-12 (p70)	−0.04	−0.08	0.06	−0.14
IL-19	−0.06	0.01	−0.10	−0.05
IL-20	−0.15	0.03	−0.38*	−0.25
IL-22	0.19	0.17	0.20	0.06
IL-26	0.15	0.26	0.24	−0.20
IL-27	−0.24	−0.26	0.18	−0.22
IL-28A/IFN-lambda2	0.07	0.15	−0.13	−0.31
IL-29/IFN-lambda1	−0.36*	−0.27	−0.48**	−0.04
IL-35	−0.03	−0.09	0.16	0.00
MMP-1	−0.15	−0.03	0.01	−0.22
MMP-2	0.07	0.16	−0.08	0.03
MMP-3	0.11	0.24	0.06	−010
Osteocalcin	0.15	0.27	0.18	−0.16
Osteopontin	0.08	0.13	0.08	−0.12
Pentraxin-3	0.06	0.07	0.06	−0.13
sTNF-R1	0.14	0.14	0.22	−0.11
sTNF-R2	0.08	0.08	0.11	−0.10
TSLP	−0.12	−0.02	0.00	−0.26
TWEAK/TNFSF12	0.02	0.07	0.13	−0.18

Significant *p*-values;*<0.05, **<0.01, ***<0.001.

## Discussion

In the present study we aimed to elucidate the acute effect of Finnish sauna bathing session in middle-aged men and women on the mobilization of immune cells and comprehensive panel of circulating cytokines and their possible interrelationships. We found that there was a general WBC mobilization effect, which was unrelated with the change in body temperature. In contrast, only three of the 37 cytokines were affected by the hot and dry sauna-induced heat stress, but many associations were observed between the change in body temperature and circulating cytokines, particularly immediately after the sauna exposure.

Heat stress poses a significant physiological challenge to the human body; however, regular sauna bathing, as a form of controlled heat exposure, has been linked to substantial health benefits [33]. This study aimed to explore potential mechanisms underlying these benefits, with a particular focus on the effects of acute sauna bathing on circulating WBCs and cytokines. As PV loss such as that induced by physical exercise [34;35] has marked influence on the concentrations of WBCs, all results in the present study were corrected for minor PV changes as heat exposure could induce PV loss. Although no systematic PV reduction was observed in response to sauna exposure (as participants were allowed to drink water ad libitum), the observed increases in hemoglobin concentration and hematocrit suggest that some PV loss did occur. Mobilization of red blood cells from storage sites such as the spleen is unlikely in humans, making hemoconcentration a plausible explanation. Therefore, the observed increases in total WBCs and subtypes such as lymphocytes and neutrophils likely reflect a genuine physiological response to heat stress – indicative of immune cell mobilization – rather than an artifact of PV loss. However, the results also indicate that the proportions of the WBCs did not change, suggesting generalized WBC response to heat stress.

Interestingly, the mobilization of WBCs into the circulation due to sauna-induced heat stress was not associated with the change in body temperature, measured from the ear in the present study, but it was rather more general and a consistent response across all subjects. It may thus be that the temperature change does not have to be very large for WBC mobilization to occur. Indeed, we did not find marked associations between the changes in WBCs and circulating cytokines. However, there were many significant associations between the changes in body temperature and changes in the cytokines, particularly immediately after the sauna session. Cytokines whose change correlated positively with the change in body temperature were IFN-a2, IFN-b, IFN-y IL-2, IL-8, IL-11, IL-12 (p40), IL-19, IL-20, IL-22, IL-28A/IFN-lambda2, IL-29/IFN-lambda1, IL-35, MMP-1, TSLP, TWEAK/TNFSF12. Furthermore, cytokines whose change correlated negatively with the change in body temperature were Chitinase 3-like1 and gp130/sIL-6Rb. As these associations were largely unaffected by further adjustment for sex and BMI, it is plausible that the associations are general heat stress responses not affected by sex and adiposity. Thus, sauna-induced heat stress with a significant change in the body temperature appears to associate and affect cytokine responses, but the physiological end-result of these responses remains to be addressed as it cannot be derived based on these observational study results.

To the best of our knowledge there are no similar studies that have addressed WBC and especially various cytokine responses to sauna-induced heat stress. However, Behzadi et al. studied certain responses and reported that IL-6 increased in response to 2 × 10 min of sauna bathing (+0.92 pg/mL), but not following the 1 × 10 min session (+0.17 pg/mL) [36]. IL1-RA increased during the 1 × 10 min (+51.27 pg/mL) and 2 × 10 min (+30.78 pg/mL) sessions, but CRP did not change in response to either sauna session [36]. Other blood biomarker responses have also been characterized. For instance in an earlier study involving 52+ years of age men and women who were non-naïve sauna users, median N-terminal pro-B-type natriuretic peptide level was 46.0 ng/L before the sauna exposure, and it increased to 50.5 ng/l immediately after the sauna (median change, + 12.00%) and remained persistent at 30-min post-sauna (From pre-sauna to post-30-min sauna, + 13.93%) [37]. Interestingly, the changes were more evident in males, but in that study there were no significant changes in overall levels of CRP, creatine kinase, high sensitivity troponin I, and creatine kinase-MBm. Nevertheless, there was a minor increase in levels of creatine kinase in males, by 3% [37]. It may be that the differences in the protocols could have affected the previous and current results. We chose the current protocol as it applies very well to the sauna bathing in Finland in real world and has also been used research-wise earlier [26,30;38]. It could also be that the responses are different in persons who do not use sauna regularly or used it seldom as compared to those persons who use sauna regularly and are thus likely more heat-adapted, but in the present study we did not find evidence that the responses, at least with the variables studied here, would differ between persons with different sauna visit habits.

The study has several strengths and limitations. While we measured key WBC subtypes, including lymphocytes and neutrophils, we did not assess monocytes, eosinophils, and basophils separately. Additionally, we did not examine immune cells in greater detail – such as specific T-cell subsets or natural killer cells – which could have provided further insights into the immune response to sauna-induced heat stress [34;39;40]. Furthermore, although we measured many cytokines in the blood circulation, we do not know the effects and possible interactions they eventually had in response to heat stress and to which tissues they were mobilized and with which cells they interacted eventually; the same applies to WBCs. Further, we do not know from which tissues WBCs would have been mobilized. Additionally, we do not claim that tympanic thermometer, which we used to measure body temperature, would measure core temperature [41;42]. It is however feasible and fast in these kind of sauna study settings with relatively large subject population. Further, although we adjusted our results by PV loss, which was not marked as subjects could drink water during and after the sauna session, it is acknowledged that PV loss is quite a typical physiological phenomenon as a response to heat stress and leads to the condition, in which concentrations of many variables increase and this is also what different receptors, cells and organs are exposed to. It remains to be determined how the concentration of the measured variables might have changed if participants had not been allowed to drink water, potentially leading to more substantial PV loss. Further, it is important to note that this study examined an acute physiological response, which does not reflect chronic resting conditions. For example, elevated WBC counts at rest are typically indicative of systemic inflammation and poor health status [43], whereas acute increases in response to a physiological stimulus – such as sauna bathing – are more likely to reflect enhanced immune surveillance and potentially beneficial health effects. Finally, it remains to be investigated whether a combination of intermittent heat and cold stress such as cold water immersion [44–46] has potential to modify these responses, both acutely and chronically.

In conclusion, this study demonstrates that a 30-minute session of acute Finnish sauna bathing induces immune cell mobilization, independent of sweat-induced hemoconcentration. Moreover, the association between changes in body temperature and circulating cytokine levels suggests that heat stress and immune activation may partly, but certainly not completely, mediate the beneficial health effects of sauna bathing.

## Abbreviations

WBCs: white blood cells,
FSB: Finnish sauna bathing,
BMI: body mass index,
MXD cells: monocytes, eosinophils, basophils
WBCs, Hb: hemoglobin,
PV: plasma volume,
CRP: C-reactive protein,
ILs: interleukines
